# Exploring the Social Validity and Diffusion Potential of Common Naturalistic Developmental Behavioral Intervention Strategies Implemented in Community Preschools

**DOI:** 10.3390/bs15030357

**Published:** 2025-03-13

**Authors:** Sophia R. D’Agostino, Trenton J. Landon, Alyssa Roylance, Avery Briggs, Naima Bhana-Lopez

**Affiliations:** Department of Special Education and Rehabilitation Counseling, Utah State University, 2865 Old Main Hill, Logan, UT 84322, USA; trent.landon@usu.edu (T.J.L.);

**Keywords:** early intervention, social validity, naturalistic developmental behavioral intervention, parent perspectives, autistic perspectives

## Abstract

It is imperative that researchers include the perspectives from key voices regarding early support practices, yet very few studies have included direct assessment of autistic individuals and parents of young autistic children. Despite emerging evidence of effectiveness of naturalistic developmental behavioral intervention (NDBI) strategies, it is currently unknown whether autistic adults and parents of autistic individuals know about NDBI and if they view NDBI strategies as socially valid practice. We aimed to explore the perceptions of autistic adults and parents of young autistic children regarding the social validity of NDBI strategies implemented in community preschool classrooms and their dissemination potential. We conducted a convergent mixed methods research design to collect quantitative survey data and qualitative semi-structured interview data. We received survey responses from 33 autistic adults and 37 parents of young autistic children and interviewed 12 autistic adults and 12 parents of young autistic children. We conducted a series of paired samples and independent samples *t*-tests to compare perceptions between groups and thematic analysis to analyze qualitative data. Results indicated high levels of social validity for NDBI across both groups of participants and the need for dissemination of NDBI. Agreement between autistic adults and parents of young autistic children on the social validity of NDBI and recommendations for dissemination are promising preliminary findings that NDBI researchers and practitioners may draw upon when engaging in collaborative support planning and participatory research efforts.

## 1. Introduction

Naturalistic developmental behavioral intervention (NDBI) is an umbrella term for an empirically validated set of supports^1^ for young children with autism that combine developmental science and behavioral science (Bruinsma et al., 2020; Schreibman et al., 2015; Sandbank et al., 2020, 2023; Tiede & Walton, 2019). NDBI were derived in part from the science of applied behavior analysis (ABA) and align with the seven dimensions of ABA; therefore, they are considered an ABA-based support (Dueñas et al., 2023). NDBIs were designed to be more naturalistic, with a focus on child-led interactions, generalization of skills, and increasing child motivation (Schreibman et al., 2015). Thus, they align well with the tenants of the neurodiversity movement (Schuck et al., 2022), developmentally appropriate practices (NAEYC, 2022), and recommended practices for early childhood special education (DEC, 2014; Siller et al., 2024).

### 1.1. Perceptions of Social Validity and Satisfaction of NDBI

Investigating the importance and acceptability of the support goals, strategies, and outcomes from the viewpoint of implementers, receivers of the support, and key stakeholders is known as social validity (Wolf, 1978). One motivation for studying social validity is to give recipients of the support a voice (Snodgrass et al., 2018). Given that the primary purpose of ABA is to affect meaningful change of functional skills, the effort to continuously seek feedback from those directly experiencing behavioral supports is essential. Although the practice of assessing social validity within the behavioral literature has increased in recent years, autistic individuals’ voices are not centered (Huntington et al., 2023, 2024). Collecting social validity data from direct consumers is critical. Yet, in the case of research with young children, it can be difficult to assess the social validity of behavioral supports. That is, researchers may not be able to rely on written or spoken language from young, direct consumers. Instead, researchers may need to rely on inclusive methods to capture perspectives, which are unfortunately underutilized (Courchesne et al., 2022; D’Agostino et al., 2019).

Researchers can also assess the perspectives of indirect consumers and members of the immediate community. The perspectives of these individuals are valuable because they can provide lived experience, which can influence the dissemination and adoption of support practices. Adoption of support practices relies not only on efficacy, but also on the attitudes of implementers and community members (Brookman-Frazee et al., 2020). Despite emerging evidence of effectiveness of NDBI (Sandbank et al., 2020, 2023; Tiede & Walton, 2019), it is currently unknown whether autistic adults and parents of autistic individuals know about NDBI and if they view NDBI strategies implemented by community preschool teachers as a socially valid practice. Therefore, this study seeks to understand the perceptions of NDBI from autistic adults and parents of young children with autism.

### 1.2. Considering the Diffusion Process of NDBI

The recent literature suggests that NDBIs should be provided in community preschool programs to support the development of young autistic children with the consensus that learning opportunities should be embedded within the child’s natural environment (Odom et al., 2021; Siller et al., 2021). While these early childhood education frameworks are compatible with NDBI, NDBI extends these frameworks by emphasizing precise operationalizations of children’s learning targets, adult instructional strategies, and criteria for implementation fidelity (Siller et al., 2024). Evidence suggests the relative advantage of NDBI strategies as well as their acceptability and feasibility to preschool teachers of young children with and at risk for autism (D’Agostino & Frost, 2024; D’Agostino et al., 2024a; Zitter et al., 2023). However, NDBIs are not implemented at a wide scale within community settings (D’Agostino et al., 2023) and preschool teachers and behavior analysts are not familiar with NDBI (D’Agostino & Frost, 2024; Hampton & Sandbank, 2022). Slow dissemination and adoption of NDBIs may be due to current gaps in researchers’ foundational knowledge that hinder the effective dissemination of the key components of early autism supports, preventing them from being widely recognized and adopted by the early childhood workforce (D’Agostino et al., 2024a). Further, the implementation of NDBIs may require the de-implementation of other approaches coupled with better incentivization of NDBIs (Pickard et al., 2024).

While numerous manualized NDBI programs exist, the eight common strategies among these manualized NDBI programs were identified by Frost et al. (2020) and described in the *NDBI-Fi*, an observational coding fidelity tool designed to rate delivery of NDBI. The identified common strategies include being face-to-face and at the child’s level, following the child’s lead, modeling and expanding language, and providing communicative temptations that lead to high-quality direct teaching episodes that are motivating and involve natural reinforcement. The unpackaging of NDBI programs in the form of the eight common strategies can support dissemination at a wider scale and allow for broader exploration of the acceptability of NDBI components (D’Agostino et al., 2024b).

Diffusion is the process of communicating an innovation through the members of a social system (E. M. Rogers, 2003). In other words, diffusion is how new supports or innovations spread among members of a community. NDBIs are not widely implemented despite evidence of effectiveness and numerous packaged and branded programs in circulation for decades. The slow rate of adoption of NDBIs may be explained by the perceived attributes of this innovation, including the relative advantage, compatibility, complexity, and observability of the NDBI. E. M. Rogers (2003) defines relative advantage as “the degree to which an innovation is perceived as being better than the idea it supersedes” (p. 229). The attribute of compatibility refers to the perceived consistency of an NDBI with existing values, past experiences, and needs of potential adopters (E. M. Rogers, 2003). The complexity of an innovation is how individuals perceive the difficulty regarding understanding and use. The observability refers to the degree to which the results of the NDBI are visible to invested parties.

Another variable that may impact the diffusion of NDBIs is the nature of the communication channels diffusing NDBIs. The use of NDBI in preschools still appears to be at the knowledge of innovation stage (i.e., NDBI is not a well-known or widely used intervention program). While the implementation of ABA-based supports for young autistic children is communicated using mass communication channels and interpersonal communication, the nature of NDBI communication is unspecified. It is known that the exchange of ideas occurs most frequently between like individuals (i.e., homogenous) while heterogenous communication between dissimilar individuals rarely occurs (E. M. Rogers, 2003). For example, information shared between parents (i.e., a homogonous group) tends to flow regularly compared to information between autistic adults and parents of young autistic children (i.e., heterogenous groups). However, diffusion needs to occur through communication channels that are somewhat heterogenous (E. M. Rogers, 2003). Therefore, it is imperative that we explore potential NDBI communication channels with autistic individuals and parents of young autistic children.

### 1.3. Previous Research

Very few studies have elicited feedback from autistic adults and parents of young autistic children regarding NDBI. One study explored the social validity of a specific NDBI (i.e., Pivotal Response Treatment) from the perspectives of autistic adults from watching videos of children engaged in a Pivotal Response Treatment (PRT) session (Schuck et al., 2022). Findings from this study suggest that numerous aspects of PRT were seen as socially valid (e.g., following the child’s lead and reinforcing attempts to communicate) while other aspects of the support goals and procedures were viewed as detrimental (e.g., emphasis on spoken language and not reinforcing every attempt to communicate). Chazin et al. (2024) surveyed autistic adults, parents of autistic children, and early childhood special educators who worked with young autistic children regarding perspectives on teaching young autistic children including developing early support goals, designing learning contexts, and selecting support procedures to reduce challenging behavior. Researchers identified recurring themes across stakeholder groups, highlighting respondents’ support for goals that promote self-determination, spending most of the time in child-led learning, and implementing antecedent supports. Respondents did not support goals that promote masking or procedures that included forms of extinction.

Another study by Pickard et al. (2024) explored the barriers related to implementing NDBI from the perspective of 18 ABA frontline and supervising clinicians through semi-structured interviews. Participants reported that they enjoyed delivering the NDBI while also identifying implementation barriers related to NDBI (e.g., the need for unlearning, more training, self-efficacy, and attitudes towards NDBI). The aforementioned studies provide valuable insight regarding the social validity perceptions of invested parties on some early autism support practices. However, the perspectives from key voices, particularly autistic adults and parents of young children who received early intensive behavioral support, are missing regarding the implementation of common NDBI strategies.

### 1.4. Current Study

Although NDBI shows promise as a socially valid support and theoretically aligns with the tenants of the neurodiversity movement, researchers have called for improvements to NDBIs to enhance their social validity (Schuck et al., 2022). Additionally, researchers have called for more research on NDBI supports implemented in natural settings by parents (van Noorden et al., 2024) and teachers (D’Agostino et al., 2024a) with a focus on diffusion and dissemination research to support early autism supports (D’Agostino et al., 2024b). Further, researchers and advocates have called for greater focus on the lived experiences of autism (DePape & Lindsay, 2016; Leadbitter et al., 2021; Tesfaye et al., 2019). To address these gaps, we collected in-depth data from a small group of autistic adults and parents of young autistic children to explore their perceptions of NDBI strategies to further understand their social validity and dissemination potential. The specific research questions we addressed included the following: (1) What are parents of young autistic children’s perceptions of the social validity of NDBI strategies (i.e., acceptability of the goals, procedures, and outcomes)? (2) What are autistic adults’ perceptions of the social validity of NDBI strategies (i.e., acceptability of the goals, procedures, and outcomes)? (3) In what ways are parents’ and autistic adults’ perceptions of the social validity of NDBI strategies similar and/or different? (4) What suggestions do parents and autistic adults have to support the dissemination (i.e., nature of communication) and implementation of NDBI strategies relative to the attributes of innovation (i.e., relative advantage, compatibility, complexity, and observability)?

## 2. Methods

### 2.1. Research Design

A convergent mixed methods research design (Creswell & Clark, 2018) was used to collect quantitative and qualitative data. Quantitative data were collected in the form of an electronic questionnaire and qualitative data were collected through semi-structured interviews. This design was chosen due to the intent of the research team to mix the results of the quantitative and qualitative data analysis for combination.

#### 2.1.1. Inclusion Criteria

Following the informed consent, participants were asked to respond to three inclusion criteria questions. Autistic adults were asked the following questions: (1) Do you self-identify as autistic, have a diagnosis of autism, or are you in the process of receiving a diagnosis of autism? (2) Are you at least 18 years old and living independently or supported in a way that you can answer the questions on this survey without support workers or family members dictating your responses? (3) Do you reside in [name of state redacted]? Parents of young autistic children were asked the following questions: (1) Do you have a child 9 years of age or younger where one of the following applies: (a) has a formal diagnosis of autism from a medical professional, (b) is in the process of receiving a diagnosis of autism, (c) is regarded by a school as having language delay, social communication needs, or social skills needs, (d) has a medical diagnosis that creates difficulty in speech and/or communication, or (e) currently in the process of determining a diagnosis or school based label reflecting language/communication or social skill deficits? (2) Does your child receive/received applied behavior analysis (ABA)-based early intervention services? (3) Do you reside in [name of state redacted]?

#### 2.1.2. Questionnaire Participants

An a priori power analysis for comparing the means of two independent samples using t-tests, a medium effect size (0.5), and *α* score of 0.05 suggested a sample size of 174, with approximately 88 in each group. As this study was considered by the researchers to be preliminary in nature, it was decided that groups of approximately 30 participants in the respective groups would suffice. The questionnaire was sent to various parent support and autistic groups/listservs inclusive of potential participants across the home state of the researchers in August of 2023. The questionnaire was started by 53 autistic adults and 65 parents and completed by a total of 33 autistic adults and 37 parents of autistic children. The group of autistic adults were predominantly female (*n* = 23, 69.7%), white (*n* = 32, 97%), and had some level of post-high school education or training (*n* = 23, 69.7%). The mean age was 25.39 years (SD = 7.92). The parental group was also largely female (*n* = 26, 70.3%), white (*n* = 32, 86.5%), and had some level of college or post-high school training (*n* = 32, 86.5%). The mean age of parents was 35.3 years (SD = 7.1). See Table 1 for additional demographic information.

#### 2.1.3. Semi-Structed Interview Participants

At the end of the questionnaire, participants were asked to provide an email address if they wanted to be contacted for an interview. Twenty-four autistic adult participants and twenty-five parent participants provided an email address and were contacted to schedule an interview. A total of 12 autistic adults and 12 parents of young autistic children responded and were interviewed.

#### 2.1.4. Reflexivity Statement

The research team was composed of 5 researchers, all working or studying in a special education and rehabilitation counseling department of a predominantly white institution in the Mountain West region of the United States at the time of this study. All authors are cisgender and use pronouns aligned with the sex assigned to them at birth. Authors one, two, and four are white and not disabled. The third author is white, disabled, and neurodivergent. The fifth author is Latina and Indian and does not identify as disabled but does have a mental health condition that some consider part of the neurodivergent spectrum.

As a research team, we acknowledge that our roles grant us power. Our aim as researchers in the field of special education and rehabilitation counseling is to use this power to enhance and support the quality of life of individuals with disabilities, their caregivers, and the professionals who serve them. Our views of autism are varied. While some of us view autism as a congenital disability, others view it as a different way of experiencing the world. However, we all agree that societal expectations and social environments are often the cause of the barriers and stigma that most autistic individuals experience. We also all promote the inclusion of all individuals with disabilities, focusing our work on accommodations and adaptations, not discrimination and exclusion. The research team held each other accountable through the data analysis process by adhering to the thematic analysis process outlined by Braun and Clarke (2006, 2017), participating in self-reflection, and by framing their thematic analysis from the standpoint that the participants were the “experts” on their lived experiences.

All authors had previous professional experience working with individuals with disabilities before their current roles in academia. We recognize that our personal and professional experiences have shaped our understanding of the field of education and how to support individuals with disabilities. As such, we also recognize that biases are unavoidable; our lived experiences and how we relate to the world may have impacted our interpretation of the findings. For example, the first and fifth authors are both board-certified behavior analysts at the doctoral level (BCBA-Ds), and the third author is a BCBA. These authors, plus the fourth author, are part of a lab focused on investigating NDBI. We all believe NDBIs are socially valid and empirically validated supports for children with socio-communicative delays. The second author did not have prior experience with NDBI and had limited experience with ABA before this study.

As researchers in this field, we acknowledge the harm that some traditional ABA-based practices have caused some individuals with disabilities, which has resulted in reported trauma. Our hope is that high-quality supports can avoid this outcome. Our aim is to provide more natural and neurodiversity-affirming practices to socio-communicative supports through NDBI. All authors are advocates for changes that will move the fields of special education, ABA, and rehabilitation counseling forward. We listen and learn from the perspectives of autistic individuals as well as the individuals who support them (e.g., caregivers, family members, educators, therapists).

### 2.2. Participant Recruitment

Prior to data collection and recruitment, this study was approved by the university’s institutional review board. Participants from both groups (i.e., autistic adults and parents) were recruited using purposive sampling (Remler & Van Ryzin, 2011). Study information was shared via two social media outlets known to include individuals with autism, two university programs for adults with disabilities, a parent listserv of a local early intensive behavioral intervention clinic, and a parent listserv of a local early childhood special education program. Recruitment materials were sent to each outlet and included information describing the purpose of the study, the researchers and their background, inclusion criteria, and a link to the electronic questionnaire in Qualtrics (Qualtrics, 2023). The contacts of each outlet were asked to reshare/resend the recruitment information 4 weeks after initial recruitment and the questionnaire remained open for two additional weeks following the reminder. Participants provided informed consent via Qualtrics and received a USD 25 electronic gift card after verification that 90% of the electronic questionnaire had been completed. Participants provided an email address at the end of the questionnaire if they wanted to be contacted for an interview. Semi-structured interview participants received a USD 25 electronic gift card following the interview.

### 2.3. Data Collection

Data reported are part of a larger study that included research questions regarding participants’ experiences and perceptions of ABA-based intervention practices that they or their child had experienced. These data are reported in a separate manuscript.

#### 2.3.1. Questionnaire

The electronic questionnaire was created based on published research, published social validity questionnaires, and commonly used demographic questions (e.g., Carter & Wheeler, 2019; Schuck et al., 2022). The questionnaire was piloted prior to distribution by five individuals who were not included in this study (i.e., early childhood special education intervention researcher, an autistic adult, a quantitative expert, a parent of a child with a disability). Revisions to the questionnaire included wording of questions for clarity, organization of questions, deleting redundant questions, and removing open-ended response options. Open-ended response options were removed to make the questionnaire simpler for participants, as the second phase of this study included a semi-structured interview.

The questionnaire began with a letter of informed consent followed by the inclusion criteria questions. Participants were told that the purpose of this study was to learn about their perspectives of early support practices for young children. In addition to the three inclusion questions, the questionnaire included seven demographic questions, two NDBI video examples implemented in community preschool settings with accompanying social validity questions (the same seven questions for each scenario), and twenty-two questions regarding perceptions of NDBI strategies.

Demographic Questions. Both groups were asked seven demographic questions focusing on race/ethnicity, gender identity, employment status, age, median household income, marital status, and level of education (see Table 1).

Example NDBI Videos and Social Validity Ratings. Participants were instructed to watch two videos of preschool teachers implementing NDBI strategies. Table 2 provides a description of each of the eight common NDBI strategies with examples from each video. The participants were *not* initially provided with the information that the preschool teachers were using NDBI strategies. The first video was 1 min 53 s in length and participants were provided with the following information: The child is 4 years old and has an identified speech and language impairment. The goal targeted in this scenario is to use verbs, nouns, and adjectives in 2–3-word sentences to communicate. This goal was created by the child’s Individualized Education Plan (IEP) team, which consisted of the child’s parent(s), teachers, and service providers. The second video was 1 min 25 s in length and participants were provided with the following information: The child is 4 years old and has an identified developmental delay. The goals targeted in this scenario include (1) making a choice between two items; and (2) requesting an item from a communication partner using multiple modalities (i.e., picture, sign, word approximation). These goals were created by the child’s Individualized Education Plan (IEP) team, which consisted of the child’s parent(s), teachers, and service providers.

After viewing the videos, participants were informed that the preschool teachers in the video examples were implementing eight specific strategies. Participants were provided with a table of strategies with a description of each (adapted from Frost et al., 2020; descriptions from Table 2). Participants were *not* informed that the strategies were NDBI strategies. For each video scenario, participants were asked to select their agreement (1 = strongly disagree to 6 = strongly agree) with the statement: This is an important goal. Participants were then asked to rate their agreement (1 = strongly disagree to 6 = strongly agree) regarding their belief that helping the preschooler in the video achieve their social communication goal could improve family relationships, friendships, the potential for employment opportunities, educational opportunities, community participation, and quality of life. Participants were then asked to rate their agreement (1 = strongly disagree to 6 = strongly agree) with five statements regarding the acceptability of the strategies.

Overall Perceptions of NDBI. On a subsequent section of the electronic questionnaire, participants were informed that the strategies were called NDBI and derived from developmental psychology and the science of ABA. With this information, participants were asked to respond to the following statement: How likely are you to recommend NDBI for use in preschool classrooms? (1 = extremely unlikely to 6 = extremely likely). Participants were then asked to rate their agreement (1 = strongly disagree to 6 = strongly agree) with six statements regarding their overall perceptions of NDBI strategies.

#### 2.3.2. Semi-Structured Interview

Semi-structured interviews were conducted within two weeks of participants’ response to the questionnaire. Interview questions were developed from the results of the questionnaire responses and included questions designed to explore participants’ perspectives on NDBI practices. Participants were emailed the interview questions and informed consent prior 5 days prior to the scheduled interview date. Interviews were conducted by the first or second author via Zoom and lasted an average of 25 min (range = 11:15–41:50). The interviews were recorded and automatically transcribed in Zoom. The transcriptions were checked and corrected if needed by watching the recordings. Transcripts were sent to participants to check for accuracy prior to data analysis; this helped ensure responses remained authentic to participants’ voice.

Participants were asked 7 questions to identify why they believed their group generally liked these practices, why they would or would not recommend them, and what changes they would suggest (using the same list of NDBI practices presented in the questionnaire). They were also asked to share their thoughts on why the other participant group generally reported positive views about NDBI and to provide recommendations on how researchers could improve the dissemination of NDBI practices and better align them with the principles of the neurodiversity movement.

### 2.4. Data Analysis

#### 2.4.1. Questionnaire Data Analysis

Data analysis was conducted using IBM SPSS Statistics software (V. 30) Demographic data were analyzed using descriptive statistics. Prior to overall data analysis, statistical tests were run to determine if the responses met assumptions of equal variance. Results from a one-way ANOVA were statistically significant for the items specific to ABA-based practices (F = 17.8, df = 1, 68, *p* < 0.001) and NDBI strategies (F = 5.17, df = 1, 68, *p* < 0.03). Additional analyses using the Welch procedure and the Brown–Forsy the procedure were similar, with both tests demonstrating statistical significance, thereby suggesting that differences were present between the two groups and their perceptions of ABA (F_asymp_ = 17.0, df1 = 1, df2 = 54.3, *p* <0.001) and NDBI (F_asymp_ = 5.2, df1 = 1, df2 = 65.2, *p* = 0.03).

As a pilot study, the sample sizes were smaller than suggested by the a priori power analysis. Hence, additional analyses were conducted to ensure assumptions of normality were met. Visual inspection of boxplots suggested a relatively normal distributional shape, with no outliers. According to Levene’s test, the homogeneity of variance assumption was satisfied for the items specific to ABA-based intervention practices scores [F(1, 68), = 9.5, *p* = 0.003] and the items specific to NDBI strategies [F(1, 68), = 1.7, *p* = 0.19]. For the analysis of each research question, a series of paired samples *t*-tests and independent samples *t*-tests were conducted. Equal variance was not assumed for one item (i.e., I would suggest the use of this intervention for children with ASD and communication goals;). A Welch’s *t*-test revealed a statistically significant difference between the parental group and autistic adults, (*t* [60.9] = 3.7, *p* = 0.01). A response rate was not calculated, as the research team had no way of knowing exactly how many potential participants were invited to participate in this study. Completion rates were noted as follows: 65 parents of autistic children started and 37 completed the parental perceptions questionnaire (56.9%); 53 autistic adults started the questionnaire with 33 completing (62.2%).

#### 2.4.2. Semi-Structured Interview Analysis

The research team used the thematic analysis procedures recommended by Braun and Clarke (2006, 2017). The six-step thematic analysis process included the following: (a) coders individually read all interview transcripts to familiarize themselves with the data and generate initial codes, (b) coders met to discuss initial codes and create a list with data extractions, (c) independent generation of initial qualitative themes and thematic map, (d) coders met to discuss individual thematic maps and devise a set of themes and a candidate thematic map, (e) coders individually read the entire data set and considered the validity of individual themes and whether the candidate map accurately reflected the meanings evident in the transcripts, and (f) writing of the final report (Braun & Clarke, 2006).

In accordance with the first three steps, four members of the research team independently reviewed, coded, and mapped the thematic elements. After these independent reviews, the team met to discuss the homogeneity of the internal fit of data within preliminary themes, as well as the external fit between themes. The goal of this first meeting was to identify appropriate terminology (themes) and their definitions to best represent the participants’ own words and achieve unanimity on the major themes (Patton, 2014). When a consensus on primary themes and their definitions was reached, the same four researchers reviewed the interview transcripts using the themes identified and then independently built individual thematic maps representing the relationship of the identified qualitative themes. These members of the research team then met again to further discuss the respective thematic maps and to build consensus on the final thematic map. Following this process helped control for interpretative validity (Altheide & Johnson, 1994).

#### 2.4.3. Data Mixing

The purposes for mixing data within this study were for development and to identify areas of convergence and divergence (Creswell & Clark, 2018; Greene, 2007). First, the results from the questionnaire responses guided the development of the semi-structured interview questions. Also, data were mixed to explore perceptions of NDBI strategies and findings were interpreted to determine areas of convergence and divergence (Creswell & Clark, 2018).

## 3. Results

**Figure 1:**
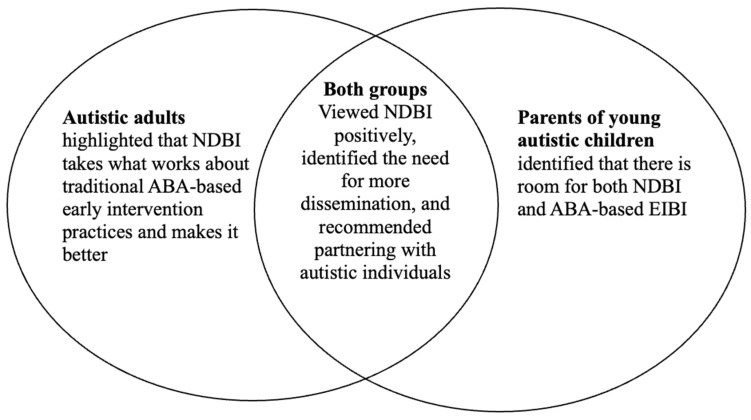
Major themes.

The results are organized by research question and are presented with quantitative results based on statistical analyses accompanied by qualitative results presented by prominent themes and representative quotes. Figure 1 diagrams the major themes and their relationship and more specific information regarding all themes and subthemes, and additional representative quotes are also provided. 

### 3.1. Perceptions of NDBI

#### 3.1.1. Social Validity Ratings Based on NDBI Videos

Participants rated the social validity of the goals, procedures, and outcomes based on two videos each depicting a preschool teacher implementing the eight common NDBI strategies with a young child with social communication goals. Results are summarized in Table 3. Both parents and autistic adults agreed that the goals targeted in each scenario were important. Both groups also rated the procedures they viewed in the videos positively and agreed that NDBI was likely to be beneficial and effective. Results indicate that parents rated statements with stronger agreement compared to autistic adults.

Participants also agreed that helping the preschooler in the video achieve their social communication goals using the NDBI strategies depicted in the video could improve family relationships, friendships, the potential for employment opportunities, educational opportunities, community participation, and quality of life (see Table 4). While both groups agreed that the child’s goal would result in improved outcomes for each item, parents rated the majority of the outcomes higher than autistic adults. Cohen’s d scores for Table 3 and Table 4 were ranged from 0.8 to 1, indicating a large effect size and further substantiating the significance of the differences in perception between the parent group and autistic adults.

#### 3.1.2. Overall Perceptions of NDBI Strategies and Comparisons Between Groups

An independent samples *t*-test was also used to determine if statistically significant differences existed between the autistic adults’ and the parents’ *overall perceptions of NDBI strategies*. Although both groups reported positive perceptions of the NDBI strategies, statistically significant differences were found between the parent group and autistic adult group with the parent group generally perceiving the NDBI practices more positively (see Table 5).

Participants were also asked to respond to the prompt: *How likely are you to recommend NDBI for use in preschool classrooms?* The responses were rated using a six-point Likert-style scale ranging from *1 = extremely unlikely* to *6 = extremely likely*. An independent samples t-test was used to compare the mean differences between the two groups (parents [*n* = 37], *m* = 5.49, *SD* = 0.9; autistic adults [*n* = 33], *m* = 4.6, *SD* = 1.2). As the groups were unequal in number and the homogeneity of variance assumption was not met (F = 4.2, *p* = 0.04), a Welch’s t-test was used to compare the results (*t* = 3.3, *df* = 58.8, *p* < 0.001). Although scores for both groups indicated a positive assessment of NDBI for use in preschool classrooms, the parent participants were more likely than autistic adult participants to recommend NDBI for use in preschool classrooms. Effect sizes for the overall perception of NDBI were also quite large (0.9 or higher), indicating a large effect size.

Qualitative results converge with quantitative results, revealing that participants’ perception of NDBI was positive. A list of themes and subthemes specifying attributes of NDBI that each participant group valued along with illustrative quotations is presented in Table 6. Attributes of NDBI that both autistic individuals and parents of young autistic children highlighted included that NDBI was perceived as natural and intuitive, and participants liked the child-led nature of NDBI. One autistic adult shared that NDBI is “a much more natural way to approach it”. A parent shared:

Well to me, so much of this is really about the interpersonal relations. When you’re with someone and you’re attuned to that person, and you’re modeling that to the child. And you’re also honoring the child by not trying to control them. You’re honoring meeting them where they are and then letting them be able to dictate some of what is happening.

Parents of young autistic children noted that there was room for both traditional ABA-based supports and NDBI. One parent explained:

I’d like to see more of the NDBI practices in more intensive ABA settings, where there can be more of, like you said, natural approaches with children, but still using ABA-based practices, because they’re children first.

Autistic adults shared that NDBI takes what works with traditional ABA-based early support and makes it better. For example, one autistic adult noted, “They’ve taken what has been shown to work with ABA, but leaving the BS. We’re leaving the bad stuff. We’re only taking the good stuff, and we’re adding a whole bunch of other good stuff”.

### 3.2. Participants’ Recommendations for Disseminating NDBI

A salient theme that emerged from interviews across both participant groups revealed the need to disseminate NDBI (see Figure 1 and Table 6). Participants were unfamiliar with NDBI; both groups shared that dissemination efforts should be focused on the knowledge and awareness levels. One parent noted:

I didn’t even know NDBI was a thing, and so maybe like that distinction of yes, it is ABA, but it’s this flavor of ABA. Maybe if we could get that out there, maybe that would be helpful and positive. Help combat some of that dentist fear that’s going on.

One autistic adult suggested effort to disseminate NDBI at the awareness and knowledge level:

So maybe just getting the word out there, because I even did a comparison. I tried Googling ABA versus NDBI, and there was a lot more information about ABA versus NDBI. And so I think just getting the word out there and maybe helping make it a more mainstream training and therapy approach. I think that would be helpful to just getting it implemented more widespread.

Both participant groups also recommended that dissemination efforts should involve partnership with autistic individuals and parents. For example, an autistic adult participant described:

Get autistic individuals in because who knows us better than ourselves? Yeah, it can be difficult to explain our experiences. It’s kind of like asking a fish what’s water like? Because this is the way we live. But we are probably the most knowledgeable about what we deal with besides the parents who raised us. And getting us more involved in that process definitely does it.

One parent suggested:

Well, I certainly think what you’re doing now, helps. And you’re listening to voices of parents who, obviously, we care about our child. We love them deeply, and we want them to be happy, healthy, and independent. But of course, adults with autism, too, who can give you like, “Hey, from me as a person, sure, maybe ABA helped me meet my milestones, but I was miserable the whole time, you know?” So definitely listening to those voices, I think, is probably the biggest and best thing you can do to improve the treatment.

Parents specifically relayed the dissemination idea of sharing positive stories and data related to NDBI use. One parent suggested:

I think showing positive stories or even positive data. I like to see data and showing that this is working. Just showing that it works. Giving graphs, or data, or anything just to show that this is the reason that we’re doing this intervention and this is how it’s helping and then allowing them to make their own changes.

Autistic adult participants suggested the use of social media and to target students and new clinicians to disseminate NDBI. One autistic adult suggested:

Put more videos out there, especially where it’s based on ABA and people already have that negative connotation. Putting the videos out there to show how it’s actually implemented and appropriate for child development, too. I think people won’t believe it until they see it. You can talk about it, but I think seeing the implementation would change a lot of people and parents’ mindsets.

## 4. Discussion

We aimed to explore the perceptions of autistic adults and parents of young autistic children regarding the social validity of NDBI strategies implemented in community preschool classrooms and their dissemination potential. Autistic adults and parents of young autistic children often bring valuable expertise to child-focused support research. Both groups can speak to what life was like for them or their child and are often drawing on a wide range of experiences due to their connections with other self-advocates and parents (Leadbitter et al., 2021). Overall, our findings indicate that both groups of participants within this study perceived NDBI strategies positively and would recommend NDBI strategies for implementation with young autistic children in community preschool classrooms. Our study confirms previous research by Schuck et al. (2022) that the social validity of NDBI models is perceived positively by autistic adults and extends this work by including the voices of parents and comparing their perspectives to autistic adults’ while examining participant perspectives of common NDBI strategies as opposed to a specific manualized NDBI. Our study also extends the work of Chazin et al. (2024) and colleagues by focusing specifically on the social validity of common NDBI strategies. Finally, our study confirms the work of Pickard et al. (2024) regarding positive perceptions of NDBI and attributes of innovation that may impact the dissemination and implementation of NDBI and extends this work by including the voices of parents of young autistic children and autistic adults.

### 4.1. Implications

#### 4.1.1. Social Validity of NDBI

We solicited feedback directly from autistic voices and parents of young autistic children by having them watch two video clips of a preschool teacher implementing common NDBI strategies in the community classroom setting. High levels of social validity for NDBI strategies suggests that both groups of participants agreed that the NDBI strategies they saw implemented in preschool classrooms were acceptable. This finding aligns with previous research indicating the potential of NDBI strategies to be socially valid (Schuck et al., 2022). Support for the social validity and recommended use of NDBI from the perspective of autistic individuals and parents of young autistic children are important findings with several implications.

Traditional forms of ABA-based support practices have received criticism for perceived dehumanizing aspects of the treatment (Anderson, 2023; Shkedy et al., 2021; Sandoval-Norton et al., 2019). Given that NDBIs are an ABA-based support viewed positively by both participant groups in this study, it would appear NDBIs are well suited as a social communication support model for young autistic children as the field of early intervention for autism strives to move toward alignment with autistic self-advocacy and the neurodiversity movement (Leadbitter et al., 2021). NDBIs are not discipline-specific and can be implemented effectively in a variety of community settings (e.g., schools, homes, clinics; Bruinsma et al., 2020) by practitioners across disciplines (e.g., teachers, behavior analysts, speech-language pathologists; S. Rogers et al., 2022; Stahmer et al., 2017) because NDBIs do not require discipline-specific knowledge for effective implementation (Ingersoll et al., 2024). These promising features, coupled with the growing evidence base for NDBIs, should be considered by practitioners when providing services to young autistic children and their families.

In the present study, statistically significant differences were noted between the parent and autistic adult groups. However, both groups largely held positive perceptions of NDBI, with parents typically demonstrating a more positive view of NDBI than the autistic adults. While both groups disagreed with the statement “NDBI strategies do result in negative side effects for children with autism”, autistic adults’ perceptions revealed they disagreed less strongly resulting in a statistically significant difference. This indicates a mistrust of many supports (even those viewed positively) by autistic adults. Given some of the larger arguments noted by autistic advocates against ABA-based supports, it would seem likely that the sense of trust between the autistic community and researchers/practitioners needs repair. Dissemination and implementation of NDBI may be a way to help bridge the apparent trust gap. Given that autistic individuals have raised concerns about ABA-based supports, dissemination of NDBI should proceed in a way that respects neurodiversity principles. Partnering with autistic individuals can support effective dissemination of NDBI.

#### 4.1.2. Diffusion Potential of NDBI

The findings from this pilot study also suggest that attributes of innovation (i.e., relative advantage, compatibility, complexity, and observability) of NDBI are met from the perspectives of autistic adults and parents of young autistic children within this study (E. M. Rogers, 2003). This finding supports the positive nature of the diffusion process of NDBI for parents of young autistic children and autistic adults. However, these are only two of the invested parties that play an important role in the dissemination and implementation of NDBI. Pickard et al. (2024) explored the perceptions of board-certified behavior analysts (BCBAs) and registered behavior technicians and identified the relative ease of NDBI (i.e., another attribute of innovation) as a barrier. Findings suggest that implementing NDBI was perceived as difficult and numerous implementation barriers were identified (e.g., the need for unlearning, more training, self-efficacy, and attitudes towards NDBI) from these influential invested parties despite these implementers feeling quite positive about incorporating NDBI into their ABA approaches. It is imperative that researchers continue to explore perceptions of NDBI across varied invested parties and focus on studying dissemination.

Additionally, our findings revealed the nature of communication that may influence the diffusion of NDBI. The two groups of participants within this study were homogeneous and highlighted the need for communication across groups involving partnering with autistic advocates and targeting parents in dissemination efforts. A heterogenous network can aid rapid diffusion (E. M. Rogers, 2003). Therefore, dissemination efforts should be made to connect influential groups and opinion leaders within groups to support the widespread dissemination of NDBI. Social media may be a promising avenue to aid rapid diffusion across groups since it can assist in creating connections across different communities, and several participants suggested the use of social media as a dissemination strategy. Targeted efforts to disseminate NDBI at the knowledge and implementation levels across invested parties (parents, autistic adults, BCBAs, etc.) could be influential in supporting the wide-spread uptake of NDBI. This could be achieved by creating training materials and opportunities for behavior analytic professionals regarding NDBI, such as classes within behavior analytic coursework or professional development opportunities on implementing the common NDBI strategies. Training modules for parents to learn about NDBI could also be created to allow for implementation and generalization at home. Continued evaluation of the implementation and diffusion of NDBI will be essential as implementation is a cyclical process and barriers to implementation may change over time.

### 4.2. Limitations

The presented findings from this pilot study should be considered bearing in mind the following limitations: First, the survey was distributed in one Mountain West state. The demographic information shows respondents were mostly white, female, and had a higher level of education. Such a sample would likely reduce the generalizability of the study’s findings to a broader, more diverse population. For more accurate and widely applicable results, a sample should ideally reflect the diversity in the larger population that the researchers intend to make conclusions about. Second, the autistic adult sample is not representative of the autistic community, nor those who receive ABA-based supports (Centers for Disease Control and Prevention, 2023; Dietz et al., 2020). Hence, our sample is not representative of all autistic adults and parents of young autistic children, so perspectives may not generalize broadly. Third, respondents had to be able to complete the survey on their own. This requirement may have limited autistic adults’ responses to those who are literate and technologically fluent. Additionally, given this requirement, these responses may not account for the perspective of those with greater communicative needs. Although it is important to hear experiences from those with more communicative repertoires, our sample is not representative of diversity within the autistic community.

### 4.3. Conclusions

Both participant groups in our study indicated a preference for NDBI strategies, which supports the growing call for wide-spread dissemination and implementation of NDBI (D’Agostino et al., 2023, 2024b; Ingersoll et al., 2024). Agreement between autistic adults and parents of young autistic children on the social validity of NDBI is a promising preliminary finding that NDBI researchers and practitioners may draw upon when engaging in collaborative support planning and participatory research efforts. Finally, this study’s findings highlight the need for the dissemination of NDBI, which could inspire training initiatives and support implementation.

## Figures and Tables

**Table 1:** Demographics.

		Autistic Adults (*n* = 33)	Parents of Autistic Children (*n* = 37)
Gender			
	Woman	23 (69.7%)	26 (70.3%)
	Man	4 (12.1%)	10 (27.0%)
	Transgender/nonbinary	5 (15.1%)	0
	Other	1 (3%)	0
	Prefer not to answer	0	1 (2.7%)
Race/Ethnicity			
	White	31 (97%)	32 (86.5%)
	Multiracial	1 (3%)	1 (2.7%)
	Hispanic or Latino	0	3 (8.1%)
	Prefer not to answer	0	1(2.7%)
Age			
	Mean/standard deviation	25.4 (7.9 SD)	35.3 (7.1 SD)
	Median	22	33
	Mode	19	27
	Range	18–33	27–55
Level of Education			
	High school graduate	10 (30.3%)	5 (13.5%)
	Some college	12 (36.4%)	2 (5.4%)
	Trade school	1 (3%)	1 (2.7%)
	Associate’s degree	3 (9.1%)	3 (8.1%)
	Bachelor’s degree	5 (15.2%)	20 (54.1%)
	Master’s degree	1 (3%)	2 (5.4%)
	Doctoral degree	1 (3%)	4 (10.8%)
Employment Status			
	Employed part time	16 (48.5%)	4 (10.8%)
	Student	9 (27.3%)	
	Employed full time	4 (12.1%)	18 (48.6%)
	Self-employed	2 (6.1%)	3 (8.1%)
	Unemployed—not looking for work	2 (6.1%)	12 (32.4%)
Combined Household Income (USD)			
	Less than 25,000	16 (48.5%)	3 (8.1%)
	25,001 to 50,000	3 (9.1%)	3 (8.1%)
	50,001 to 75,000		10 (27.0%)
	75,001 to 100,000	2 (6.1%)	11 (29.7%)
	100,001 to 150,000	1 (3%)	6 (16.2%)
	150,001 or more	1 (3%)	4 (10.8%)
	Prefer not to answer	10 (30.3%)	

**Table 2:** Descriptions and examples of NDBI common strategies.

NDBI Strategy	Description	Example
Face-to-face and on child’s level	The child’s and adult’s bodies are oriented toward each other, and they are at a similar level. Adult is within the child’s line of sight.	Video 1: The adult kneels down next to and facing child. Video 2: The adult sits across from and facing the child.
Following the child’s lead	Adult provides several developmentally appropriate activity options and allows the child to choose which toy or activity to play with, how to play, and how long to stay with an activity. The adult then joins in the child’s chosen activity.	Video 1: The child is engaged with magnets and the adult joins. Video 2: The child is engaged with toy cars and the adult joins.
Displaying positive affect and animation	Adult displays rich positive affect, matched to the child’s sensory needs, to promote child engagement. This may include adjusting vocal quality or tone, gestures, and facial expressions.	Video 1: The adult uses an excited voice to match the child’s excitement. Video 2: The adult laughs with the child when he pushes the car down the ramp.
Modeling appropriate language	Adult adjusts language to the child’s developmental level; most utterances match the child’s current abilities, while others are slightly above a child’s current ability. The adult avoids asking questions or giving commands (outside of direct teaching episodes) and primarily comments.	Video 1: The adult repeats “It’s not balancing, it’s floating” after child’s approximation. Video 2: The adult narrates “The red car. Put it on” after child chooses red toy car.
Responding to attempts to communicate	Adult verbally responds to the child’s attempts to communicate, including vocalizations, eye contact, word approximations, gestures, joint attention, etc.	Video 1: The adult hands the child another magnet when he says “more”. Video 2: The adult accepts child’s pointing to which car they want. Video 2: The adult says “go” and allows the child to push the car after child approximates “go”.
Using communicative temptations	Adult deliberately creates situations meant to elicit communication from the child. This may involve blocking the child’s play, putting toys in sight but out of reach, limiting or withholding access to toys, using toys or containers for which the child needs assistance, or modeling a silly or unusual play act.	Video 1: The adult holds the magnets away from the child. Video 2: The adult blocks child from pushing the car.
Pace and frequency of direct teaching episodes	The adult directs the child to demonstrate new or emerging skills by giving an instruction or cue. There is at least a brief period of time between direct teaching episodes in which the child receives access to a reinforcer, and the adult leaves space for child initiations.	Video 1: The adult waits for the child to play with a magnet, then directs them to ask for more. Video 2: The adult waits for the child to play with a car before directing them to choose another.
Quality direct teaching episodes	Adult uses clear teaching opportunities that are developmentally appropriate and motivating. The adult supports a correct response and provides contingent natural and social reinforcement.	Video 1: The adult targets a several-word request from the child who uses phrases. Video 2: The adult targets a one-word response from a child who has some single words. Video 2: The adult targets driving a toy car from a child with simple functional play skills.

Note. Descriptions adapted from (Frost et al., 2020).

**Table 3:** Autistic adults’ and parents of autistic children’s social validity ratings of NDBI videos.

	Autistic Adults		Parents of Autistic Children				
	*m*	SD	*m*	SD	*t*	*df*	Cohen’s *d*
I find NDBI strategies appropriate for young children with autism	4.8	1.1	5.4	0.9	2.6 *	68	1
NDBI strategies are effective in increasing the skills of young children with autism	4.8	0.9	5.4	0.9	2.7 **	68	1
I would suggest the use of NDBI strategies to others	4.6	1.2	5.4	0.9	3.1 **	68	1.1
NDBI strategies do result in negative side effects for children with autism.	3.2	1.2	2.4	1.5	−2.3 *	68	1.3
NDBI strategies are an acceptable way to target the child’s skills.	4.6	1.3	5.4	0.9	3.2 **	68	1.1
I like the procedures used in NDBI.	4.8	1.2	5.2	1.1	1.5	68	1.1
Overall, NDBI strategies are beneficial to young children with autism.	4.9	1.1	5.5	0.9	5.6 *	68	1

Note. * α ≤ 0.05; ** α ≤ 0.005; 1 = strongly disagree to 6 = strongly agree.

**Table 4:** Perceived outcomes based on video examples 1 and 2.

Video	Item Prompt: The Goal Will Improve:	Parents		Autistic Adults		df	*t*	Cohen’s *d*
		*m*	*SD*	*m*	*SD*			
#1	Family relationships	5.5	0.9	5.1	0.7	68	2.0 *	0.8
	Friendships	5.6	0.9	5.2	0.7	68	1.8 *	0.8
	The potential for employment opportunities	5.5	1.1	5.1	0.8	68	1.7 *	0.9
	Educational opportunities	5.6	0.9	5.0	0.8	68	2.6 **	0.9
	Community participation	5.4	0.9	40.9	0.8	68	2.0 *	0.9
	Overall quality of life	5.5	0.9	40.9	0.9	68	2.7 **	0.9
#2	The goal will improve							
	Family relationships	5.5	0.9	40.9	0.7	68	2.7 **	0.8
	Friendships	5.5	0.9	5.1	0.8	68	2.0 *	0.9
	The potential for employment opportunities	5.4	1.1	5.1	0.8	68	1.1	0.9
	Educational opportunities	5.4	1.2	5.0	0.9	68	1.5	1
	Community participation	5.2	1.3	40.9	0.8	68	0.9	1
	Overall quality of life	5.5	0.9	40.9	1.0	68	2.8 **	0.9

Note. * α ≤ 0.05; ** α ≤ 0.005; 1 = strongly disagree to 6 = strongly agree.

**Table 5:** Overall perceptions of NDBI strategies and comparisons between groups.

Item In Both Videos:	Parents		Autistic Adults		df	*t*	Cohen’s d
	*m*	*SD*	*m*	*SD*			
I find this intervention acceptable.	5.5	0.9	4.9	0.9	68	2.7 *	0.9
I would suggest the use of this intervention for children with ASD and communication goals.	5.6	0.9	4.7	1.1	60.1	3.6 *	1
I believe this intervention is likely to be effective.	5.5	1	4.9	1	68	2.8 *	0.9
I support the continued use of this intervention in the preschool classroom.	5.6	0.9	4.9	1	68	3.2 *	0.9
I like the strategies used in this intervention.	5.6	0.9	4.9	1	68	3.1 *	0.9

Note. * α ≤ 0.005; 1 = strongly disagree to 6 = strongly agree.

**Table 6:** Themes and subthemes with illustrative quotations.

Themes and Subthemes	Illustrative Quotations
Positive perception of NDBI (both groups) Natural and intuitive, child-led (both groups) Kind, fun, respectful, promotes generalization, builds real communication (autistic adults) Catered to the individual, kids liked it, good teaching and parenting practice, developmentally appropriate, builds confidence, feels easier/accessible, positive adult and child interaction (parents of young autistic children)	“I think it’s a more gentle way of coaxing behavior, bringing someone out rather than chastising them. I think just being very open to the ways in which the child is attempting to communicate or perhaps acknowledging why they’re not wanting to communicate or interact”. (Autistic adult) “And it’s fun. Like it’s fun for kids to play and learn through play”. (Autistic adult) “Well to me, so much of this is really about the interpersonal relations. When you’re with someone and you’re attuned to that person, and you’re modeling that to the child. And you’re also honoring the child by not trying to control them. You’re honoring meeting them where they are and then letting them be able to dictate some of what is happening”. (Parent of young autistic child) “I feel like these NDBI practices are almost building a friendship with the student or with the person that they’re working with”. (Parent of young autistic child)
Takes what works with ABA approaches and makes it better (autistic adults)	“I think that it’s trying to take what’s good about ABA and integrate it with a more easy to accept to the person who is undergoing it. It’s trying to make it not so traumatic”. (Autistic adult)
Room for both NDBI and traditional ABA approaches (parents of young autistic children)	“But I don’t understand how you could definitely say that one is better than the other, especially since they’re based within each other, from ABA and NDBI practices. And so I would like to continue to focus both”. (Parent of young autistic child)
Dissemination of NDBI is needed at the knowledge and awareness level (both groups) Targeting students and new clinicians (autistic adults) Using social media (autistic adults) Positive stories (parents of young autistic children)	“So just more learning for the parents, whether it’s through pamphlets, or if they have just online information that’s more appropriate, would help, in my opinion”. (Parent of young autistic child) “creating the awareness and the opportunities to see it and understand” (Autistic adult) “Starting with the newbies, the fresh ones. The young ones who are stepping into this field and getting it into the curriculum is going to be your best way of getting things changing and fixed”. (Autistic adult) “I think it’s always easier to judge something when you have exposure to it. Like if you’ve only heard something is bad and you’ve never seen it, what are you going to think? You’re probably gonna think it’s bad. But if you have a chance to see it and make your own informed opinion, it might be different than what everyone else has told you”. (Parent of young autistic child)
Partner with autistic individuals (both groups)	“Unfortunately, yeah, you’re going to have push back, especially from those adults who went through that. But also showing the proof of hey- This is actually what we’re doing now. We really do want your opinion on this. We are trying to work. It may take us a while for us to build that trust because it takes us a while to build trust sometimes, after being burned so many, many times. We can do it, and we want to be involved. That is the biggest thing. Get us involved”. (Autistic adult) “So ask the adults with autism, *Well, what would you change?* or *How do we make this better?* and letting them manipulate a little bit”. (Parent of young autistic child)

## Data Availability

The data presented in this study are available on request from the corresponding author due to ethical restrictions.

## Note

1 To shift away from a medical model of disability, it is more appropriate to use the term “support” instead of “intervention”, with the exception to reference of NDBI and questionnaire items.
